# Isolation and Characterization of Lytic Properties of Bacteriophages Specific for *M*. *haemolytica* Strains

**DOI:** 10.1371/journal.pone.0140140

**Published:** 2015-10-09

**Authors:** Renata Urban-Chmiel, Andrzej Wernicki, Diana Stęgierska, Marta Dec, Anna Dudzic, Andrzej Puchalski

**Affiliations:** Sub-department of Veterinary Prevention and Avian Diseases, Institute of Biological Bases of Animal Diseases, Faculty of Veterinary Medicine, University of Life Sciences, 20–033, Lublin, Poland; ContraFect Corporation, UNITED STATES

## Abstract

**Aim of Study:**

The objective of this study was isolation and morphological characterization of temperate bacteriophages obtained from *M*. *haemolytica* strains and evaluation of their lytic properties *in vitro* against *M*. *haemolytica* isolated from the respiratory tract of calves.

**Material and Methods:**

The material for the study consisted of the reference strain *M*. *haemolytica* serotype 1 (ATCC^®^) BAA-410™, reference serotypes A1, A2, A5, A6, A7, A9 and A11, and wild-type isolates of *M*. *haemolytica*. Bacteriophages were induced from an overnight bacterial starter culture of all examined *M*. *haemolytica* strains treated with mitomycin C. The lytic properties and host ranges were determined by plaque assays. The morphology of the bacteriophages was examined in negative-stained smears with 5% uranyl acetate solution using a transmission electron microscope. The genetic analysis of the bacteriophages was followed by restriction analysis of bacteriophage DNA. This was followed by analysis of genetic material by polymerase chain reaction (PCR).

**Results:**

Eight bacteriophages were obtained, like typical of the families *Myoviridae*, *Siphoviridae* and *Podoviridae*. Most of the bacteriophages exhibited lytic properties against the *M*. *haemolytica* strains. Restriction analysis revealed similarities to the P2-like phage obtained from the strain *M*. *haemolytica* BAA-410. The most similar profiles were observed in the case of bacteriophages φA1 and φA5. All of the bacteriophages obtained were characterized by the presence of additional fragments in the restriction profiles with respect to the P2-like reference phage. In the analysis of PCR products for the P2-like reference phage phi-MhaA1-PHL101 (DQ426904) and the phages of the *M*. *haemolytica* serotypes, a 734-bp phage PCR product was obtained. The primers were programmed in Primer-Blast software using the structure of the sequence DQ426904 of reference phage PHL101.

**Conclusions:**

The results obtained indicate the need for further research aimed at isolating and characterizing bacteriophages, including sequence analysis of selected fragments. Moreover, standardization of methods for obtaining them in order to eliminate *M*. *haemolytica* bacteria involved in the etiopathogenesis of BRDC is essential.

## Introduction

*Mannheimia haemolytica* is the primary etiological agent of bovine respiratory disease complex (BRDC) in cattle and sheep, and exists as commensal flora in the upper respiratory tract of ruminants. It is responsible for significant economic losses to the livestock industry [1]. *M*. *haemolytica* is a common pathogen isolated from more than 80% of cases of bovine respiratory disease complex (BRDC) in cattle and exerts a synergistic effect with such respiratory viruses as BRSV, PI-3 and BHV-1. During the respiratory syndrome these bacteria play a crucial role in generalization of the disease process, also known as acute fibrinosuppurative and necrotizing inflammatory pulmonary infections.

The widespread use of antibiotic therapy and metaphylaxis (mainly in the USA, Canada and Australia) has led to the emergence of bacteria characterized by multiple antibiotic resistance, which substantially reduces the effectiveness of therapy and the elimination of pathogens. Due to the limited possibilities for combating bovine respiratory disease complex, alternative methods are sought, among which high hopes are placed in treatments involving the use of bacteriophages specific for bacterial pathogens isolated from the respiratory tract of cattle. A positive element in contrast to other methods used in alternative therapies is the common occurrence of bacteriophages, which are present in wastewater, water bodies, soil, forest undergrowth, and food products. They are estimated to number about 10^31^, which is 10 times greater than the number of bacteria that have been characterized. This is a significant factor facilitating both their acquisition and characterization of their suitability for fighting bacterial infections [2, 3].

The effectiveness and safety of phage therapy is partly due to the specificity of phages for selected bacteria, manifested as the ability to infect only one bacterial species, serotype or strain. Such a mechanism does not destroy commensal flora, and due to self-replication of bacteriophages during treatment they do not need to be applied repeatedly. However, this mechanism is mainly characteristic of temperate phages [3].

Bacteriophages were obtained from *M*. *haemolytica* for the first time in the 1950s [4]. It was then suggested that all *M*. *haemolytica* strains belonging to biotype A serotype 1 exhibit specificity for induction of phage φPhaA1 [5]. A study by Froshauer et al. [6], analysing 14 *M*. *haemolytica* strains, confirmed the presence of prophages with genome size of about 40 kb. In the early 21st century, Highlander et al. [7] demonstrated that phages isolated from *M*. *haemolytica* serotypes A1, A5, A6, A9 and A12 contain a sequence similar to that of phage P2, φMhaA1-PHL101. Furthermore, Davies and Lee [8] analysed 15 *M*. *haemolytica* strains isolated from cattle and 17 obtained from sheep and confirmed the presence of prophage genomes ranging in size from 22 to 45 kb [9]. Characterization of bacteriophages that can be used in treating BRDC is also based on their lytic properties against tested bacteria. Hsu et al. [9] did not obtain strictly lytic bacteriophages capable of lysis of *M*. *haemolytica* strains isolated from the upper respiratory tract of cattle. The bacteriophages obtained had lysogenic properties, which significantly impeded their use in fighting infections induced by strains of *M*. *haemolytica*. Hence comprehensive characterization combined with an evaluation of genetic and phenotypic properties seems to be an essential element of the search for other bacteriophages with lytic properties against these bacteria.

The objective of our study was the isolation and morphological characterization of bacteriophages obtained from *M*. *haemolytica* strains and evaluation of their lytic properties *in vitro* against reference and wild type isolates of *M*. *haemolytica*.

## Material and Methods

### Bacterial strains and growth conditions

Our material for the study consisted of the reference strain *M*. *haemolytica* serotype 1 (ATCC^®^) BAA-410™, reference serotypes A1 (P588), A2 (499), A5 (P501), A6 (6174), A7, A9 and A11, and wild type isolates of *M*. *haemolytica* obtained from cattle with respiratory syndrome– 25, 99, 101, and 1480 (all of these isolates belonged to serotype A1). The reference serotypes were obtained from Prof. Schimmel of the Bundesinstitut für gesundheitlichen Verbraucherschutz und Veterinärmedizin, Jena. The wild-type strains were obtained from cattle with BRDC in Poland.

Bacteria were stored at −85°C in 50% (v/v) glycerol in brain heart infusion broth (BHIB; Sigma) followed with 0.5 mL of filtered (0.45 μm and 0.22 μm Millipore filters) nasal swabs obtained from calves. Isolates were plated onto blood agar (BHIA) containing 5% (v/v) sheep’s blood and incubated overnight at 37°C. The starter cultures were prepared by inoculating a few colonies from the agar plates into 25 mL volumes of BHIB and incubating them overnight at 37°C with shaking at 120 r.p.m [8].

### Ethics Statement

For collection of *M*. *haemolytica* strains from cattle the authors obtained the guidelines of the Second Local Animal Care Ethics Committee in Lublin (Approval numbers 39/2009, 09 June 2009).

### Induction of bacteriophages

Bacteriophages were induced according to Davies & Lee [8]. Briefly, 20 mL volumes of pre-warmed BHIB in 100 mL Erlenmeyer flasks were inoculated with 0.2 mL of an overnight bacterial starter culture of each isolate of *M*. *haemolytica* suspended in medium and incubated at 37°C with shaking at 120 rpm. During the logarithmic growth phase of the bacteria (about 4 h) mitomycin C was added to a final concentration of 0.3 mg/mL and incubation was continued for a further 12 h. The optimum concentration of 0.3 mg/mL was determined in preliminary experiments in which concentrations ranging from 0.01 to 5.0 mg/mL were compared. The induction of phages (bacterial cell lysis) was monitored by measuring the optical density (OD 660 nm) after the addition of mitomycin C and in the control (growth of bacteria without mitomycin C). The phage particles were separated from the cells by centrifugation of the cultures at 3,000 g for 20 min at 4°C and filtration of the supernatants through 0.45 μm Millipore filters. Lytic properties and host ranges were determined by plaque assays. The lytic titre was evaluated by the dilution method in SM buffer [10]. Before further characterization the phages were individually plaque-purified three times on agar plates according to Han et al. [11]

### Morphological analysis by electron microscopy

Bacteriophage morphology was examined in negative-stained smears with 5% uranyl acetate solution using a transmission electron microscope [12]. Five millilitres of each phage suspension in TM buffer were adsorbed onto a glow-discharged carbon-coated 200 mesh copper grid. The phage particles were negatively stained with 5% uranyl acetate solution (pH 4.0) for approximately 1 min and examined with a Zeiss LEO 902 electron microscope at an acceleration voltage of 80 kV and a magnification range of 20,000–250,000.

### Bacteriophage host range

The host range of induced bacteriophages was determined by plaque assay as follows. Lawns of indicator strains (all strains used in this study) were prepared as overlays by adding 100 mL of overnight cultures to 4 mL of molten soft agar (0.7% agar, BTL, PL) and 2 mM CaCl_2_ in BHI broth at 45°C. After mixing, these were immediately poured onto a base layer of BHI agar and the plates were air-dried in a laminar flow hood for 20 min at room temp. Five microlitres of each filtered phage suspension was spotted onto plates seeded with each indicator strain. The plates were incubated overnight at 37°C and examined for zones of lysis (plaques).

### RE analysis of bacteriophage DNA

Bacteriophage DNA was obtained from 5 mL of filtered phage suspensions with the Wizard lambda DNA purification kit (Promega, PL), according to the manufacturer’s instructions. RE analysis of bacteriophage DNA was carried out with double-digest enzymes *Hind*III (1.3 μl) and *Cla*I (1.3 μl, Fermentas, Lithuania) and single digestion with TaqI (1.3 μl, Fermentas, Lithuania). The reactions were incubated at 37°C for 5 h and approximately 40 ng digested phage DNA fragments were separated by 4% polyacrylamide gel electrophoresis and stained with ethidium bromide (8 mg/l) according to the manufacturer’s instructions.

### PCR-analysis

The PCR mix consisted of 2.5 μl of 10X DNA polymerase buffer, 1 μl of 10 mM dNTP (nucleotides) mix (Fermentas, Lithuania), 1 μl of 5 mM solution of each primer composed in Primer-BLAST on the basis of the complete sequence of bacteriophage phi-Mha A1-PHL101 (ATCC BAA-410) (yielding a product of 734 bp), 1 μl of thermo-stabile RED TaqTM DNA polymerase (Fermentas, Lithuania), and 16.5 μl DNA-RNA free water (Sigma-Aldrich, Ge). The mixture was supplemented with sterile water to a final volume of 25 μl. The primers used for amplification of phage DNA fragments were 5’- GAC GGC CAC GAC AAA GCC GA -3’ and 5’-ACC GCT TGC CTG GGT TGA GC—3’ (Blirt, PL). For the PCR reaction, denaturation was conducted for 5 min at 95°C and 35 cycles were carried out in the following conditions: denaturation for 30 s at 95°C, hybridization for 45 s at 62°C, and elongation for 1 min at 72 C. The elongation step after the last cycle was prolonged to 8 min. PCR products were evaluated in 1.5% agarose gel stained with ethidium bromide. The products obtained were analysed using Quantity One software (Bio-Rad, USA).

The PHL101 *M*. *haemolytica* phage was used for comparison because the *M*. *haemolytica* strains A1, A5, A6, A9 and A12 contained sequences of the P2-like phage φMhaA1-PHL101. Furthermore, *M*. *haemolytica* serotypes A1, 2, A5 and A6 are most often isolated from the upper respiratory tract of cattle with respiratory syndrome [7, 9].

Cluster analysis was shown as a tree diagram using Statistica 10.0 (StatSoft, USA).

## Results

**Fig 1:**
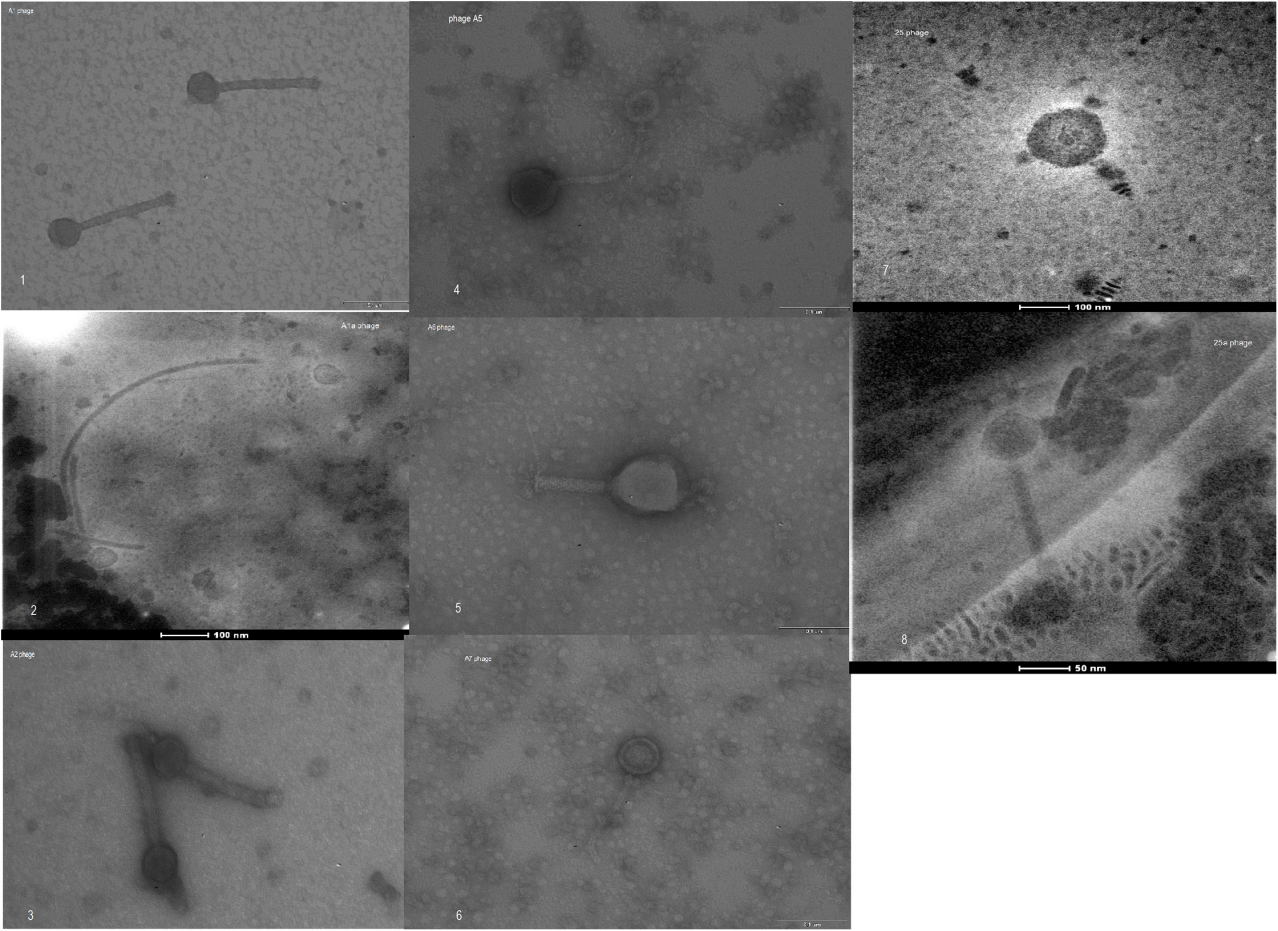
Negative-stained electron micrographs of bacteriophages induced in isolates of *M*. *haemolytica* (1–8). Legend:*Myoviridae*-type phage (1, 3, 5, 8); *Siphoviridae-*type phages (4, 6); *Podoviridae*-like phage (7). [Numbers in brackets indicate the number of the image].

**Fig 2:**
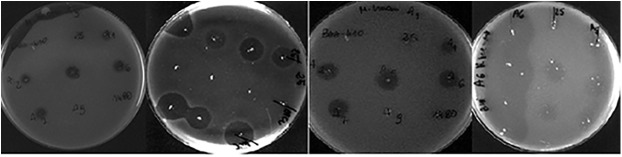
Lytic zones (plaques) of bacteriophages specific for *M*. *haemolytica* strains.

Eight bacteriophages were obtained in our study, which were characterized microscopically and identified on the basis of morphological structure as belonging to the families *Myoviridae*, *Siphoviridae* and *Podoviridae* of the order *Caudovirales* (Fig 1). Most of the bacteriophages exhibited strong lytic properties characterized by the formation of plaques in the form of clear bacterial growth inhibition zones on double-layer plates with BHI agar (Fig 2). Only two of the phages obtained, φ1a and φ25a, exhibited no typical lytic properties, which were observed in the form of types of plaque on top agar (turbid due to growth of lysogenic cells), even after 5-fold purification.

The bacteriophages we obtained were characterized by a broad spectrum of lytic activity against the analysed strains of *M*. *haemolytica* of the reference serotypes A 1, 2, 5, 6, 7 and 11 and wild type isolates no. 25, 99 101 and 1480 (Table 1). The broadest spectrum of activity against the analysed *M*. *haemolytica* isolates was observed in the case of phages φ5 and φ6. A similar spectrum of antibacterial activity was also observed in the case of phages φ2, φ7 and φ25 (Table 1).

**Table 1:** Types, titres and lytic activity spectrum of bacteriophages specific for some strains of *M*. *haemolytica* serotypes.

Phage no.	Morphology	Serovar *M*. *haemolytica*	Lytic titre	Spectrum of lytic activity
φ1	*Myoviridae*	1	10^−8^	BAA-410, 1, 2, 7, 9, 101, 1480
φ1a	*Siphoviridae*	1	10^−7^	BAA-410, 1, 2, 7, 9, 101
φ2	*Myoviridae*	2	10^−6^	1, 2, 7, 9, 99, 101, 1480
φ5	*Siphoviridae*	5	10^−6^	BAA410, 1, 2, 5, 7, 9, 25, 99, 101, 1480
φ6	*Myoviridae*	6	10^−8^	BAA-410, 1, 2, 5, 7, 9,25,99 101, 1480
φ7	*Siphoviridae*	7	10^−7^	1, 2, 5, 7, 9, 25, 99, 101, 1480
φ25	*Podoviridae*	25	10^−7^	1, 2, 5, 7, 9, 25, 99
φ25a	*Myoviridae*	25	10^−7^	1, 2, 5, 7, 9, 25, 99, 101, 1480
PHL-2	*Myoviridae*	BAA- 410	10^−6^	BAA-410, 1, 2, 5, 25

**Fig 3:**
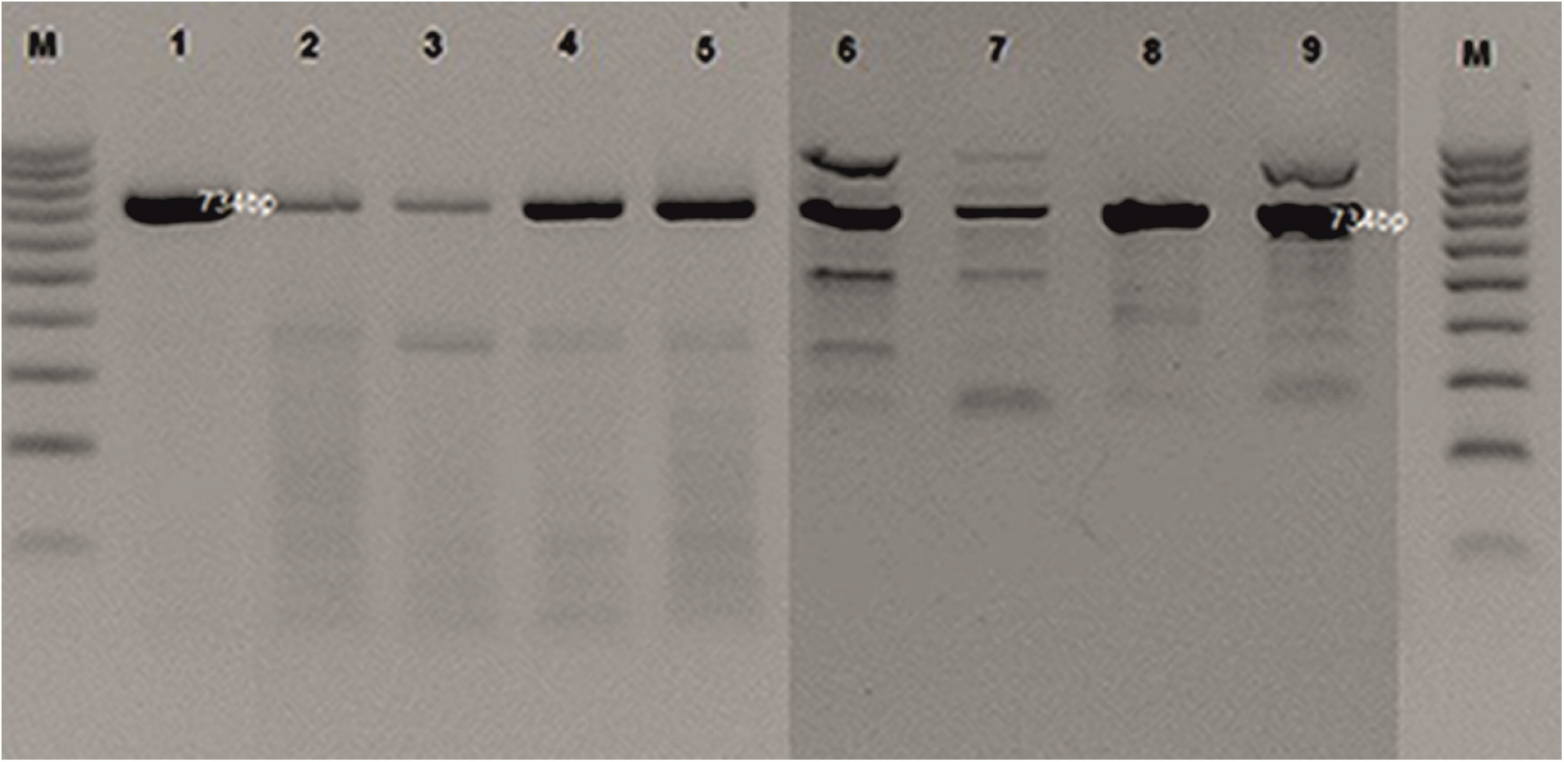
PCR analysis profiles of phage DNA from *M*. *haemolytica* isolates (lanes 1–9). Legend: φPHL-1 (from strain BAA-410); φ25; φ25a; φA1; φA1a; φA6; φA5; φA7; φA2; DNA markers (100–1000 bp, Fermentas) are shown as lanes (M).

In the analysis of the PCR products for the reference P2-like phage phi-MhaA1-PHL101 (DQ426904) and the phages of all the *M*. *haemolytica* serotypes analysed, a 734-bp product was obtained. In the case of phages φA6, A5 and A7, additional fragments of >900 bp to 1,000 bp were observed as well. Moreover, in the profiles of all the phages additional bands of ≤500 bp were present (Fig 3).

Restriction analysis of genomic DNA made it possible to determine similarities to the reference P2-like phage obtained from strain ATCC *M*. *haemolytica* BAA-410.

**Fig 4:**
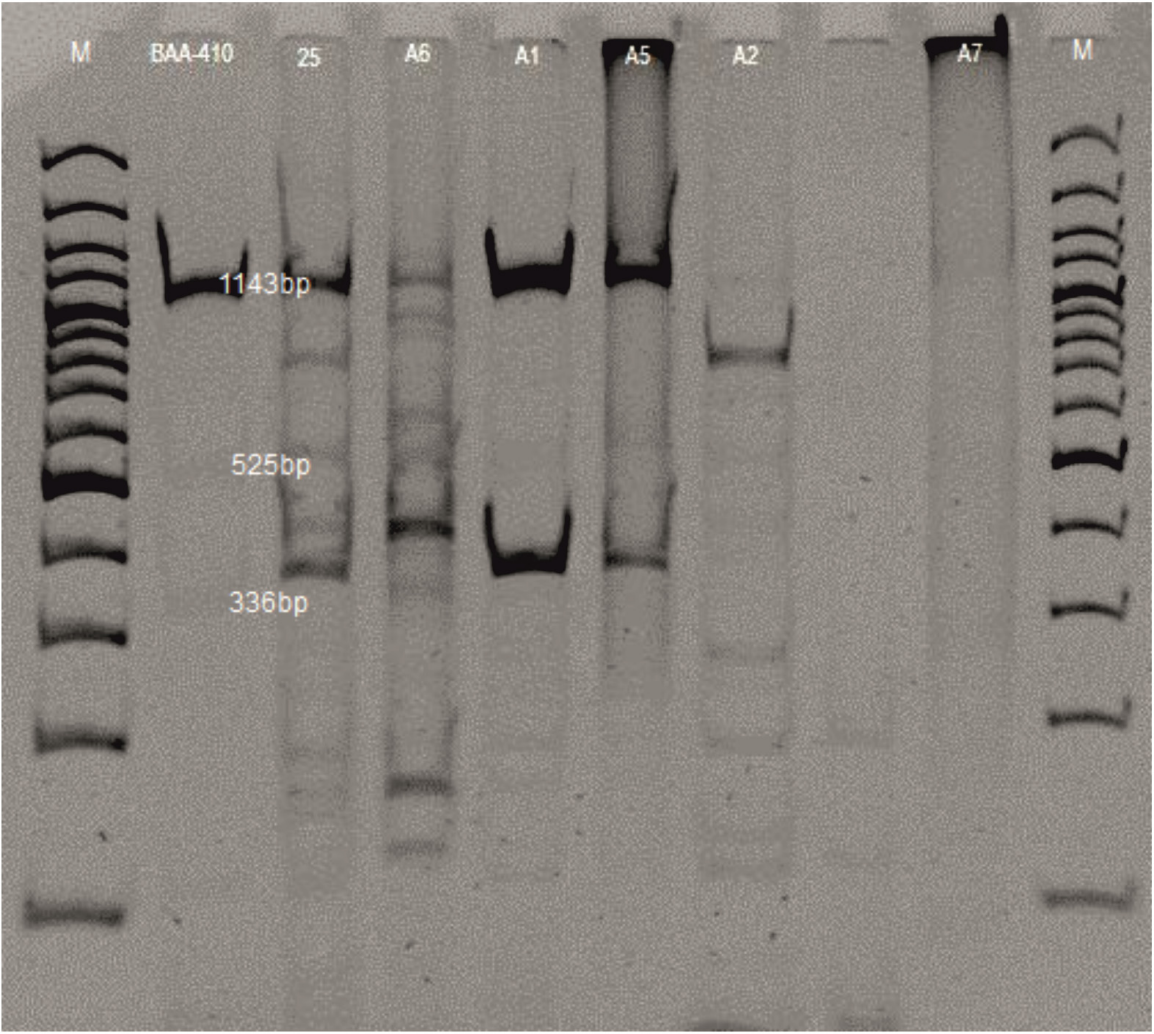
Restriction analysis profiles of phages from *M*. *haemolytica* isolates; double-digested with *Cla*I and *Hind*III. Legend: φPHL-1 (from strain BAA-410); φ25; φA6; φA1; φA5; φA2; φA7; M—DNA markers (100–3000 bp, Fermentas) are shown as lanes.

**Fig 5:**
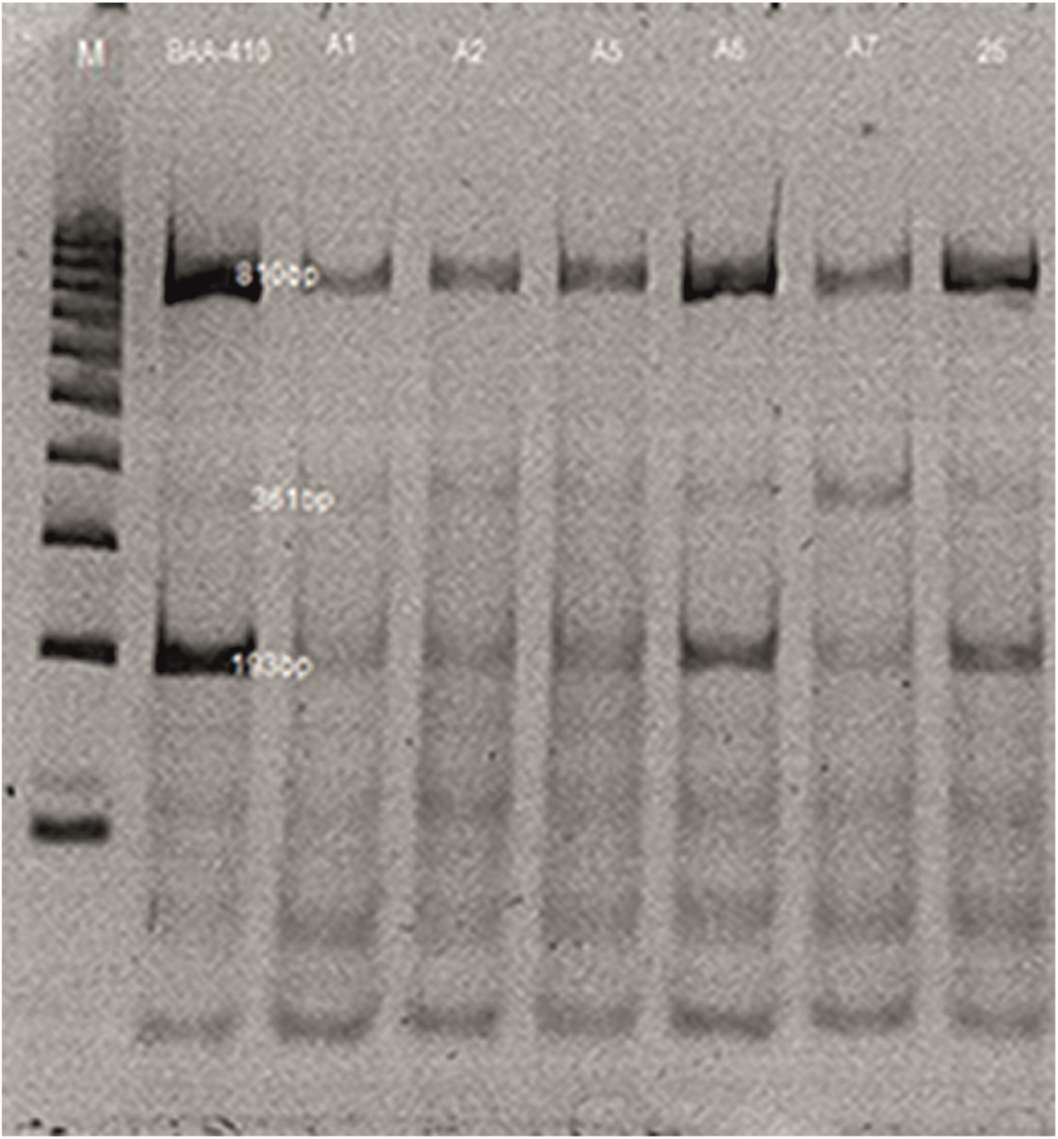
Restriction analysis profiles of phages from *M*. *haemolytica* isolates single-digested with TaqI (lines 1–7). Legend: φPHL-1 (from strain BAA-410); φA1; φA2; φA5; φA6; φA7; φ25; M—DNA markers (100–1000 bp, Fermentas).

The electrophoretic profiles of the bacteriophages following digestion with the enzymes *ClaI* and *HindIII* contained from 4 to 10 restriction fragments ranging from 114 to 1,418 bp. The P2-like bacteriophage was characterized by a band of high intensity, 1,150 bp in size, and two smaller bands with specific weights of 525 and 336 bp. A restriction fragment of 1,150 bp was also present in the profiles of phages φ1, 5, 6 and 25, but was not observed in the profile of phage φ2. In the case of phage φ7, application of *ClaI* and *HindIII* did not result in a restriction cut (Fig 4). The most similar profiles were observed in the case of bacteriophages φA1 and φA5. All of the bacteriophages obtained were characterized by the presence of additional fragments in the restriction profiles with respect to the reference P2-like phage (Fig 4). Analysis of the genetic material of the phages digested with TaqI showed no significant differences in the restriction products which would enable differentiation of the phages. All of the profiles contained a main restriction fragment 810 bp in size and smaller fragments ranging from 66 to 350 bp. It should be emphasized that the phages analysed were varied in terms of morphology (Fig 5).

**Fig 6:**
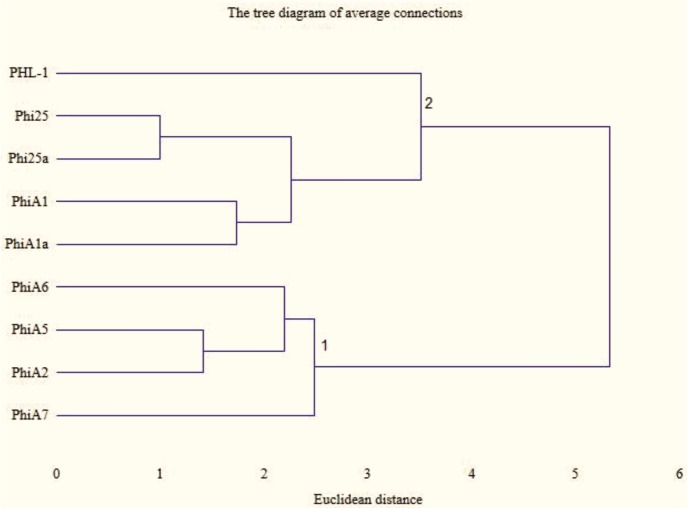
Cluster analysis tree diagram of PCR profiles of phage DNA from *M*. *haemolytica* isolates (lanes 1–9). Legend: φPHL-1 (from strain BAA-410); φ25; φ25a; φA1; φA1a; φA6; φA5; φA2; φA7; DNA markers (100–1000 bp, Fermentas, Li) are shown as lanes (M).

In the evaluation of the similarity of the profiles obtained in the PCR reactions two clusters were obtained, as well as two phages that did not belong to either cluster, i.e. the profiles obtained for the phage (PHL1-like) obtained for the reference strain *M*. *haemolytica* BAA-410 and the phage obtained from strain A7 (Fig 6). Within cluster 2 very high similarity is exhibited by phages φ25 and 25a, with Euclidean distance equal to 1.1, and phages φA1 and A1a, with Euclidean distance equal to 1.41. In the first cluster high similarity is observed for phages φA5 and φA2, with Euclidean distance of 1.0 (Fig 6).

## Discussion

In the present study we obtained bacteriophages specific for the *M*. *haemolytica* reference strains from the University of Jena and the selected field strains isolated from cases of BRDC in Poland. It should be emphasized that these bacteria were resistant to antibiotics, such as ampicillin, enrofloxacin, tilmicosin, tetracycline, and amoxicillin with clavulanic acid [13].

The lytic effect of the bacteriophages observed against the analysed strains of *M*. *haemolytica* is indicative of certain differences with respect to the properties that had previously been obtained in bacteriophages specific for *M*. *haemolytica*. The latest research [11] also has not obtained ‘strictly’ lytic phages infecting *M*. *haemolytica* strains isolated from naso-oesophageal secretions of cattle. The authors report that none of the phages obtained induced uncontaminated (clear) bacterial growth inhibition zones (plaques).

The results obtained indicate that these phages with a lytic cycle could occur as a result of keeping the *M*. *haemolytica* strains in the medium supplemented with filtered nasal swabs obtained from the upper respiratory tract of calves. It is also likely that some of them are phages originating in the animals’ housing. However, in another study [14] in which phages for *S*. *aureus* were induced with mitomycin C, lysogenic phages were obtained, as well as one lytic phage.

In the present study we found that the bacteriophages obtained exhibited lytic properties against the *M*. *haemolytica* strains tested. In addition to phages exhibiting a lytic cycle, phages with lysogenic properties were obtained as well, but because they had little effect in eliminating bacteria, these bacteriophages were not used for further analysis. According to Gill & Hyman [15], one method of increasing the likelihood of isolating bacteriophages with lytic properties is to combine material collected from the respiratory tracts of different animals.

The bacteriophages obtained in the present study we classified according to their morphological structure as belonging to the families *Myoviridae*, *Siphroviridae* and *Podoviridae*, thereby confirming results published by Davies & Lee [8], which were the first, and until now the only results indicating the possibility of inducing several phages belonging to different families from the same host. Diverse morphological structure is also indicated by the analysis of the genetic material of the phages, in restriction analysis and PCR. However, in the case of use of the enzyme TaqI, no differences were found in the restriction profiles obtained from individual bacteriophages, and due to the significant differences in the results obtained, research must be continued. In a study by Froshauer et al. [6], as a result of induction of phages from 14 strains of *M*. *haemolytica* with mitomycin C, in all cases the authors observed the same restriction enzyme digestion pattern of phage DNA, corresponding to a genome of about 40 kb. Based on their results, the authors suggest that all strains carried the same prophage and that this phage was widely distributed among *M*. *haemolytica*.

Analysis of the degree of similarity between the bacteriophages obtained, based on cluster analysis, showed the greatest similarity in the case of bacteriophages isolated on the same strain (this applies to phages φ25 and 25a and to φA1 and A1a). Substantial similarity was also observed in the case of phages isolated on different serotypes of *M*. *haemolytica* strains, A5 and A2.

As suggested by Hsu et al. [9], the dominant *M*. *haemolytica* serotypes with bacteriophage-inducing properties are serotypes A1, 2 and 6. In the present study, apart from these serotypes, bacteriophages were also obtained from serotypes A5 and 7, which were characterized by lytic properties against the *M*. *haemolytica* serotypes tested, which will undoubtedly further knowledge in this area.

To sum up, the results of the present study indicate that phage induction is possible in the examined strains of *M*. *haemolytica*. The phages obtained demonstrated diversity in electron microscopy and RE analysis. The observed lytic properties of the phages can be the basis for further research concerning their possible use for eliminating *M*. *haemolytica* in cattle.

However, these results require further research aimed at isolation and comprehensive characterization of bacteriophages, including sequence analysis of selected fragments. It is also essential to optimize methods for acquiring phages in order to standardize them for possible use in eliminating *M*. *haemolytica* bacteria involved in the etiopathogenesis of BRDC in cattle.

## References

[1] Rice JA, Carrasco-Medina L, Hodgins DC, Shewan PE. Mannheimia haemolytica and bovine respiratory disease. Anim Health Res Rev 2007; 8: 117–128. 10.1017/S1466252307001375 18218156

[2] Brüssow H, Canchaya C, Hardt WD. Phages and the evolution of bacterial pathogens: from genomic rearrangement to lysogenic conversion. Microbiol Mol Biol Rev. 2004; 68: 560–602 15353570 10.1128/MMBR.68.3.560-602.2004 PMC515249

[3] Brüssow H, Kutter E. Chapter 6 “Phage Ecology “in Bacteriophages: eds. Kutter E., Sulakvelidze A. Bacteriophages Biology and Applications pp. 129–163, CRC Press Boca Raton, Fla, USA 2005.

[4] Saxena SP, Hoerlein AB. Lysogeny in Pasteurella: I. Isolation of bacteriophages from Pasteurella strains isolated from shipping fever and those from other infectious processes. J Vet Res Mhow 1959; 3: 53–66.

[5] Richards AB, Renshaw HW, Sneed LW. Pasteurella haemolytica bacteriophage: identification, partial characterization and relationship of temperate bacteriophages from isolates of Pasteurella haemolytica (biotype A, serotype 1). Am J Vet Res 1985; 46: 1215–1220. 4003898

[6] Froshauer S, Silvia AM, Chidambaram M, Sharma B, Weinstock GM. (Sensitization of bacteria to danofloxacin by temperate prophages. Antimicrob Agents Chemotherap. 1996; 40: 1561–1563 10.1128/aac.40.6.1561 PMC163371 8726041

[7] Highlander SK, Weissenberger S, Alvarez LE, Weinstock GM, Berget PB. Complete nucleotide sequence of a P2 family lysogenic bacteriophage, φMhaA1-PHL101, from M. haemolytica serotype A1. Virology, 2006; 350: 79–89. 16631219 10.1016/j.virol.2006.03.024

[8] Davies RL, Lee I. Diversity of temperate bacteriophages induced in bovine and ovine *Mannheimia haemolyica* isolates and identification of a new P2-like phage. FEMS Microbiol Lett. 2006; 260: 162–170. 16842340 10.1111/j.1574-6968.2006.00314.x

[9] Hsu YH, Cook SR, Alexander TW, Klima CL, Niu YD, Selinger LB, et al Investigation of *Mannheimia haemolytica* bacteriophages relative to host diversity. J Appl Microbiol. 2013; 114: 1592–1603. 10.1111/jam.12185 23489937

[10] Golec P, Wiczk A, Łoś JM, Konopa G, Węgrzyn G, Łoś M. Persistence of bacteriophage T4 in a starved *Escherichia coli* culture: evidence for the presence of phage subpopulations. J Gen Virol. 2011; 92: 997–1003. 10.1099/vir.0.027326-0 21177930

[11] Han JE, Kim JH, Hwang SY, Choresca CH Jr, Shin SP, Jun W, et al Isolation and characterization of a Myoviridae bacteriophage against Staphylococcus aureus isolated from dairy cows with mastitis. Res Vet Sci. 2013; 95:758–763. 10.1016/j.rvsc.2013.06.001 23790669

[12] Xie H, Zhuang X, Kong J, Ma G, Zhang H. Bacteriophage Exc-A is an efficient therapy for *Escherichia coli* 3–1 caused diarrhea in chickens. J Gen Appl Microbiol. 2005; 51: 159–163. 16107753 10.2323/jgam.51.159

[13] Wernicki A, Puchalski A, Urban-Chmiel R. Antibiotic susceptibility and plasmid profiles P. haemolytica strains. Med Weter. 1999; 55: 623–626.

[14] Matsuzaki S, Yasuda M, Nishikawa H, Kuroda M, Ujihara T, Shuin T. et al Experimental protection of mice against lethal *Staphylococcus aureus* infection by novel bacteriophage fMR11. J Infect Dis. 2003; 187: 613–24. 12599078 10.1086/374001

[15] Gill JJ, Hyman P. Phage choice, isolation and preparation for phage therapy. Current Pharm Biotechnol 2010; 11: 2–14. 10.2174/138920110790725311 20214604

